# Supplementary figures and images for: Development of a seroepidemiological tool for bat-borne and shrew-borne hantaviruses and its application using samples from Zambia
Source: PLoS Negl Trop Dis. 2024 Nov 21;18(11):e0012669. doi: 10.1371/journal.pntd.0012669 (PMC11651553; doi:10.1371/journal.pntd.0012669)

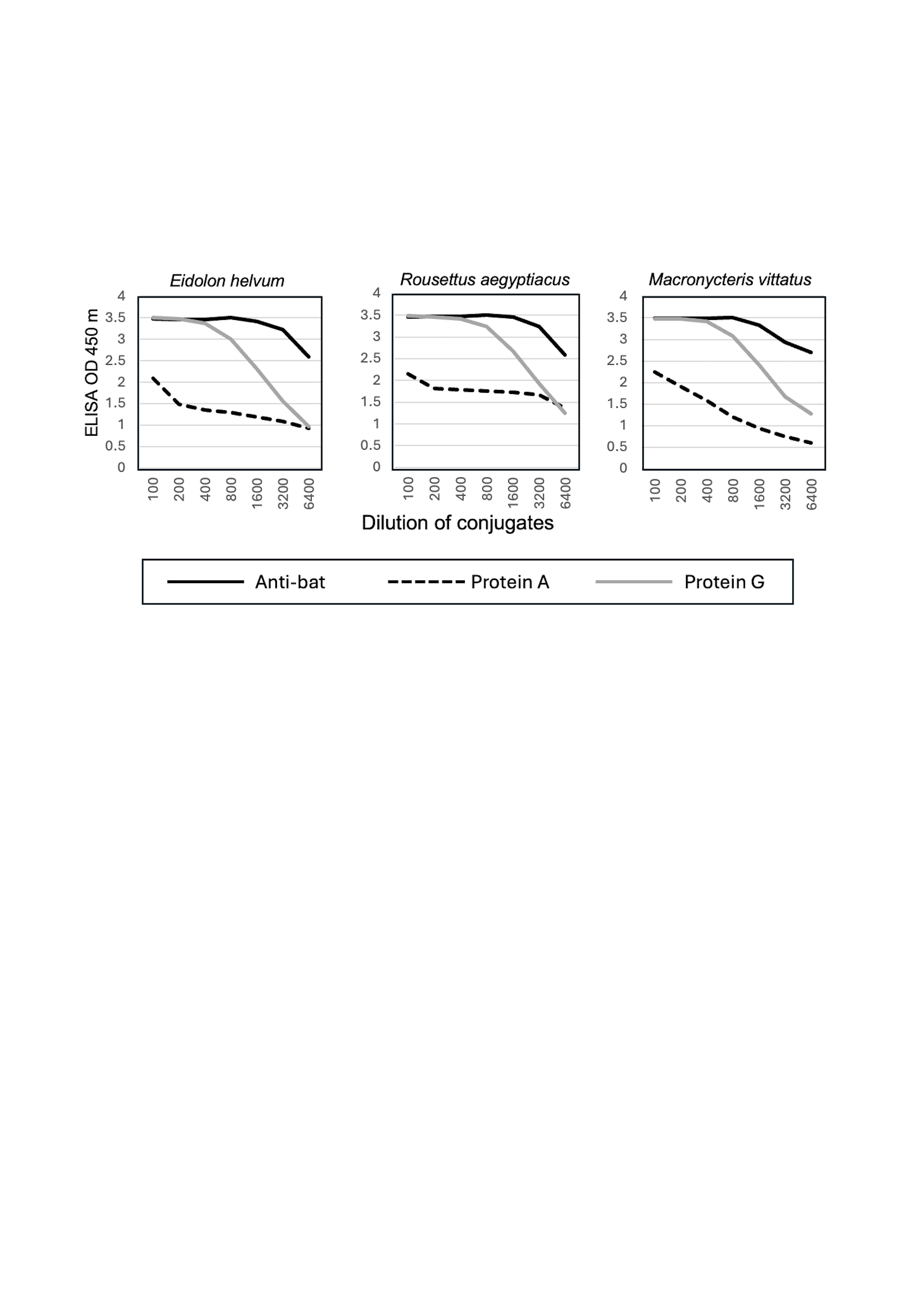

Supplement: S1 Fig — [39]. Briefly, bat serum was diluted with phosphate buffered saline at 1:1000 and absorbed to 96-well ELISA plate at 4C overnight. After blocking with bovine serum albumin, serially diluted conjugates were applied to wells. (TIFF) [file pntd.0012669.s001.tiff]
