# Supplementary material for: Development of a seroepidemiological tool for bat-borne and shrew-borne hantaviruses and its application using samples from Zambia
Source: PLoS Negl Trop Dis. 2024 Nov 21;18(11):e0012669. doi: 10.1371/journal.pntd.0012669 (PMC11651553; doi:10.1371/journal.pntd.0012669)
Supplement: Attachment — Submitted filename: Sikatarii et al. comments.docx [file pntd.0012669.s005.docx]

PLoS NTD comments:

The manuscript by Sikatarii et al aims to develop a novel tool for screening sera from bats and other species for anti-hantavirus antibodies. Given that there is a large knowledge gap with respect to the prevalence and distribution of bat-borne hantaviruses as well as shrew-borne hantaviruses, there is a need for these types of approaches and tools. The authors were able to express the N proteins of 5 bat- and 6 shrew-borne hantaviruses in VeroE6 cells and screen bat serum samples for antibodies reactive against these proteins. The authors made some interesting discoveries that will require further investigation and significant effort put into field studies and wildlife surveillance to expound upon. They have developed an interesting approach that should become a useful tool for hantavirus researchers. I have a few comments that should be addressed.

The section of the methods for the secondary Ab for bat IgG starting at line 204 through line 212 is the exact same as the section in the results from 289-297. The section of the results should be changed to provide a description of what was found, i.e. that the ODs were higher at greater dilutions for the Ab compared with protein A and G as shown in the figure. Also the ELISA should be described, at least in some detail to explain that reactivity against 3 bat species was tested and how those reactivities compared (for example in line 208 – reactive against what?). While this is partly addressed in the results and discussion later, what is the actual target of the commercial anti-bat IgG? Is there a predicted species reactivity of this Ab and how cross-reactive is it? While the Ab was “more reactive” against the 3 species tested, how broadly true is this? At lesser dilutions (around 1:400), protein G was equivalent, and possible more broadly cross-reactive than the anti-bat Ab. Would using protein G at 1:400 have allowed for better detection of bat Ab in your assays? This should be considered or at least commented on. Running a comparison would be worthwhile to see if any possible positive would be missed, especially if a broader range of species are to be tested in the future.

With respect to the initial IFAT methodology (presented in Fig 2A) and used throughout, simply having the negative control consist of untransfected VeroE6 cells and unimmunized serum is not sufficient. Using Fig 2A as an example, the results presented are clear, however additional controls should be added. This includes serum from unimmunized mice bound to transfected cells (all antigens) to show that there is no non-specific binding leading to fluorescence in your assay. And sera from immunized mice bound to untranfected cells will show that there is no non-specific binding of immune sera to VeroE6 cells that do not express hantavirus N antigens. Also, the use of the monoclonal as a positive control against these antigens would be beneficial. As the data are currently presented, these cannot be ruled out.

Minor comments:

Line 209: delete “IgG” after Goat in Goat anti-bat IgG

Line 249: the authors state that there is diffused fluorescence in the cytoplasm, which they may be. Why did they not use DAPI to stain the nuclei? This would provide clear evidence that staining is localized in the cytoplasm and is usually standard for these types of IFAT.

Line 271: in this section it is not clear what cut-off the authors used for determining what was positive cross-reactivity. In the discussion it mentions a possible 4800 dilution as positive, but it again isn’t clear as this is mentioned as a requirement for Western blot. They should clarify that here and, in the methods, because there are multiple instances of sera reacting at 3200 dilution in Table 1, which might confuse the reader when the authors mention only the ROBV and QZNV cross-reactivity. It also mentions there is “almost” one-way cross-reactivity for ROBV and QZNV, but this is also not clear. Does that mean that 6400 almost falls below the cut-off?

Lines 307-314: the authors us acronyms for the viruses throughout the paper but then switch to virus names in this section. They should keep this consistent throughout.

Line 396: the low cross-reactivity mentioned here is, I assume, related to the above comment, where some of the samples were positive at 3200. The authors do a good job of explaining why this might be here, but as with the above comment, I think a quick explanation of the chosen cut-off in the results and methods would make things clear earlier in the paper to the reader.

Line 416: this wording is a bit confusing. It should maybe read “IFA antibody titers above 4800”.

Line 428: it should be made clear this is purely speculation, that insectivorous bats may be exposed through a different route.

Line 434: the numbers here, 80 and 97%, are not correct. This is not the positivity rate reported. I assume they mean the 97 and 80 absolute numbers reported in table 3?

Line 548: the authors state that the study investigated bat- and shrew-borne hantaviruses circulating in Zambia. This is not really the case, and the wording should be changed. There was no active surveillance of bats or shrews carrying virus in the study, only retrospective analysis of serum to examine possible exposures in convenience samples.

Line 465: I would also change this sentence, as this study does not really clarify the distribution of these viruses in Zambia. First, it does not look at the presence of viruses, or even RNA, so there is no data on the presence of either type of virus, only past exposure across a (long) given time period. Second, there is no data presented on the “distribution” of the viruses or seroprevalence in Zambia, as no data are given on the locations of the animals sampled, only that the animals sampled were from Zambia.

Line 466: all that can be concluded from the data presented is that bats harbor antibodies that are reactive to shrew-borne viruses, not that bats have acquired the viruses. While the nice data presented here suggest minimal cross-reactivity, this was only for 5 or 6 viruses of each virus.

Line 468: relatedly, this phenomenon should be discussed further, specifically how if we are able to consistently find reactive Ab in bats to “shrew-borne” viruses (or viruses that were initially discovered in shrews), their labelling as “shrew-borne” may be a misnomer. We would need studies to look at the actual prevalence of virus or RNA in bats vs shrews of these viruses.

Figure 2: there should be labels for each of the individual antigens added to Fig 2A.
