# Supplementary material for: Development of a seroepidemiological tool for bat-borne and shrew-borne hantaviruses and its application using samples from Zambia
Source: PLoS Negl Trop Dis. 2024 Nov 21;18(11):e0012669. doi: 10.1371/journal.pntd.0012669 (PMC11651553; doi:10.1371/journal.pntd.0012669)
Supplement: Attachment — Submitted filename: Review for PLoS NTD.docx [file pntd.0012669.s006.docx]

The manuscript “Development of a seroepidemiological tool for bat-borne and shrew-borne hantaviruses and its application using samples from Zambia” by Sikatarii et al. is an interesting paper that describes the development of a serological testing algorithm and suggests that “shrew-borne” hantaviruses more commonly infect bats than “bat-borne” hantaviruses. The sampling appears to be opportunistic from previously available samples so is lacking the numbers needed for extensive analysis (this is not necessarily a negative but the bat capture study does not appear to have the statistical power for confident modeling) but is convincing to show the utility of the seroepidemiological tool. There are several confusing or missing information in the paper which needs to be clarified prior to submission (see specific comments below).

In the Abstract and author summary.

Line 38- please clarify which positive control antiseta (i.e. mouse and rabbit) were generated.

Line 39 – please clarify that the authors evaluated the reactivity on mixed transfections and on singly transfected Vero cells. Perhaps also include a justification as to why vero E6 cells were chosen as they are poorly transfectable.

Line 42 – the word altogether means completely or totally, where are the authors appear to mean “all together” as in at the same time.

Lines 56-58 – I am unsure what the authors are trying to say here. I do not know howthe sentiment of “more data has been generated than there are people to make meaningful conclusions from” is relevant to a paper which provides more data and a tool to generate additional data. I recommend removing it.

Line 64 – Remove the “therefore” from this line.

Line 65 – Remove the word “some” and change the word “bats” to “bat”

Line 66 - Replace the “as well as” with the word “with”

Line 68 – Remove “the relevant”

In the main body of the paper:

Line 79- Change from “Apart from rodent-borne hantaviruses…” to “Apart from in rodents…”

Line 94 – Is there a reason Quezon and Robina viruses instead of QZNV and ROBV? If not change it back for consistency with the rest of the paper.

Lines 116-118 – This sentence is confusing please change it to something like “ Additionally, due to a lack of available tools, only a few small scale studies have been reported on the potential for bat- and shrew-borne hantaviruses to infect humans.”

In the materials and methods section the source of materials is not consistent, sometimes it is just the company name, sometimes it’s the company name, city, state, country, sometimes the product number is specifically listed). Make this consistent with PLoS reporting requirement.

Lines 144-160. Can the authors state how many wells were considered for each positive or negative. E.g. was each sera applied to 3 wells of the slide glass? Was it 4 wells? The N value is missing. The western blots were loaded in quadruplicate which makes me suspect it was done in n=4 throughout but this is not documented.

Similarly, were these experiments just done once or were they repeated with data from a representative repeat shown?

For the western blotting section please add the anti-Nus western blot data. This is mentioned previously in the preparation of antisera to rN proteins, however the western blot methods are missing.

Line 252 – do you have any more specific data than “approximately 10% to 20%” was it 15±5% for an N=96?

Line 253-254 – in the materials and methods the authors state that they used the anti-nus antibodies for determination of expression of recombinant antigens but in the results and in the figure it appears as though the authors are using the mouse/rabbit sera for detection of the antigen. Can you please clarify which sera were used for the assessment of protein expression.

Line 271 QZNV was accidentally written as QUZV and needs to be changed back.

Once again write in the N that these titrations are based on.

Lines 291-292 – Not sure they product numbers are listed here and nowhere else in the paper.

Lines 302-304 – Can you please explain why you only tested further the 401 that were positive for either the bat or shrew borne hantaviruses but not both? This part is confusing as it would be interesting to see information for these if it is available.

Table 2 – The math is off for this whole table. For example a total of 986 Ei helvum were tested, 270 were positive to either antigen, 81 to both antigens and 716 were negative i.e. 270 positive (for either or both) and 716 negative. However columns next to it clearly state that 156+195 =351 total bats were positive for bat or shrew antigen of which 81 were positive for both so 270 were positive for either antigen but not both. However 986-351 is only 635 negative samples instead of 716. So which number is incorrect or which title is incorrect? Please fix throughout the table.

Lines 306-340 – Typical reaction patterns of bat serum in IGAT and western blot

Why were these bats chosen to be representative? Are they from different species or all from one species? To me ZFB09-ZFB14 suggests “Zambian fruit bats” caught in 2009 through 2014 with the –xx being the number that was caught, however this leads to more questions that this study is willing to or able to explain. Please remove this nomenclature.

Figure 4. B Do the authors have data for the other bats or was titration only done on these 6 bats? If so why were these 6 bats chosen and not the other 395 bats? This really should be a stand alone table and not part B of figure 4.

D, E and F. Why are these here? Figure 4 and Figure 5 show the same things just with different antigens. Please remove these as they do not add anything beyond Figure 4A and C.

Line 345 the 25 is a typo here according to Table 3 this should be 15.

The bat sera showed reactivity to more than 1 hantavirus (71 of the 201 for bat borne and 7 of the 307 for the shrew borne) can you add another column which states which were the bat positive sera.

Lines 351 to 373. This section seems weak in its predictive power due to the lack of consistent numbers in samples.

Furthermore the text lacks any numbers, can the authors provide some data in this paragraph instead of just saying it is significant. For example Figure 6b appears to show that 11% of ALTV positivity in R. aegyptiacus compared to 6% ASAV is significantly different. But what does mean in terms of absolute numbers 41/370 for ALTA vs 22/370 for ASAV? Please include some numbers in the text.

I’m not sure what Figure 6 is trying to prove due to the inherent issues of comparing between bat species.

I would remove this section because it does not add anything to what the main point of this paper seems to be i.e. the IFAT using rNs, but not that bat capture field studies from 2008-2014 didn’t capture enough bats to reach statistical significance for a certain bat species.

Lines 375- 382 should be moved to the individual antigen screen section after line 350 as they seem to be a continuation of this section not a standalone section.

Lines 405-409. Is there any data for this in the results or mention of it in the materials and methods that I am missing? This whole section is not explained at all, why or how this was done. Remove it or explain it in methods, supplemental methods or results.

Line 414 – Replace “solely” with “individually”.

Line 415 – please add this data i.e. how many samples reactivity to 2 or 3 samples in Table 2.

Line 416 – 4800 IFA points is an impressive number but this information on the N of each analysis is lacking in the methods and results. Please add it there not in the discussion.

Line 434 Why is the total number from table 2 now listed at 80% and 97%? I was under the impression that 97/1764 samples were positive for BRNV and 80/174 were positive for QZNV. Is this what the authors mean?If it was 80% and 97% that would be very high but the data in previous sections does not seem to support this, unless the authors can explain where 80% and 97% come from.

General point – perhaps the authors want to provide a paragraph as to how they envision their tool will assist the further research that is required to advance the understanding of the epidemiology of hantaviruses.
