# Supplementary material for: Development of a seroepidemiological tool for bat-borne and shrew-borne hantaviruses and its application using samples from Zambia
Source: PLoS Negl Trop Dis. 2024 Nov 21;18(11):e0012669. doi: 10.1371/journal.pntd.0012669 (PMC11651553; doi:10.1371/journal.pntd.0012669)
Supplement: Attachment — Submitted filename: Responces to reviewers.docx [file pntd.0012669.s007.docx]

**Answers to Reviewer #1**

Manuscript Number: **PNTD-D-24-01168,** Sikatarii et al.

Development of a Seroepidemiological tool for bat-borne and shrew-borne hantaviruses and its application using samples from Zambia.

I greatly appreciate your thoughtful and helpful suggestions. I am returning the above manuscript and have made appropriate revisions based on your comments. Our responses to your suggestions are numbered according to your suggestions. Corresponding sites were shown in yellow markers in the revised manuscript.

**Answer to comments**

Reviewer 1: The manuscript by Sikatarii et al aims to develop a novel tool for screening sera from bats and other species for anti-hantavirus antibodies. Given that there is a large knowledge gap with respect to the prevalence and distribution of bat-borne hantaviruses as well as shrew-borne hantaviruses, there is a need for these types of approaches and tools. The authors were able to express the N proteins of 5 bat- and 6 shrew-borne hantaviruses in VeroE6 cells and screen bat serum samples for antibodies reactive against these proteins. The authors made some interesting discoveries that will require further investigation and significant effort put into field studies and wildlife surveillance to expound upon. They have developed an interesting approach that should become a useful tool for hantavirus researchers. I have a few comments that should be addressed.

The section of the methods for the secondary Ab for bat IgG starting at line 204 through line 212 is the exact same as the section in the results from 289-297. The section of the results should be changed to provide a description of what was found, i.e. that the ODs were higher at greater dilutions for the Ab compared with protein A and G as shown in the figure. Also the ELISA should be described, at least in some detail to explain that reactivity against 3 bat species was tested and how those reactivities compared (for example in line 208 – reactive against what?). While this is partly addressed in the results and discussion later, what is the actual target of the commercial anti-bat IgG? Is there a predicted species reactivity of this Ab and how cross-reactive is it? While the Ab was “more reactive” against the 3 species tested, how broadly true is this? At lesser dilutions (around 1:400), protein G was equivalent, and possible more broadly cross-reactive than the anti-bat Ab. Would using protein G at 1:400 have allowed for better detection of bat Ab in your assays? This should be considered or at least commented on. Running a comparison would be worthwhile to see if any possible positive would be missed, especially if a broader range of species are to be tested in the future.

Thank you for the attention drawn to the similarity in lines 204-212 and 289-297 in the original manuscript. According to your suggestion on proteins A, G and HRP-conjugated Goat anti-Bat IgG ELISA reactivities as well as the actual target of the commercial anti-Bat-IgG, this section of the manuscript has been modified as Page 11, lines 260 - 270.

“The reactivity of horseradish peroxidase (HRP)-conjugated Protein A and G (Thermo Fisher Scientific) and HRP-conjugated Goat anti-Bat IgG (H+L) (Cat: A140-118P; Bethyl Laboratories. Inc., Montgomery, Texas, USA) were compared using ELISA as previously described [39]. Serially diluted sera from three bat species (*Macronycteris vittatus, Eidolon helvum, and Rousettus aegyptiacus*) were coated onto an ELISA plate and blocked. Respective HRP-conjugated proteins were used as secondary antibodies and OPD substrates for the detection. Anti-bat IgG was more reactive against the Bat IgG from the three species tested. (Supplementary Figure S1); hence, we purchased unlabeled Goat IgG anti-Bat IgG (H+L) (Bethyl Cat: A140-118A) and 0.5 mg the IgG fraction was conjugated with Alexa 488 dye using an Alexa Fluor 488 protein labeling kit (Invitrogen, Thermo Fisher Scientific). The conjugated anti-Bat IgG served as a secondary antibody for IFAT screening of bat serum samples at a dilution of 1:1000.”

what is the actual target of the commercial anti-bat IgG?

According to the manufacturer, by immunoelectrophoresis and ELISA this antibody reacts specifically with Bat IgG and with light chains common to other Bat immunoglobulins.

Is there a predicted species reactivity of this Ab and how cross-reactive is it? While the Ab was “more reactive” against the 3 species tested, how broadly true is this?

According to the manufacturer, the anti-bat IgG may cross-react with IgG from other species. “The company confirms reactivity to bat genus species *Pteropus vampirus, Desmodus rotundus, Eptesicus fuscus, Tadrida pumila, T. condylura, Hypsignathus monstrosus, Rosettus aegyptiacus, Epomorphus crypturus, Molossus species, and Phylostomus species.* “ Page 28, lines 584 - 587 in the revised manuscript. Since this manuscript is the first of its kind in this area of scientific research, subsequent studies may give more information on its broad application in the future.

Would using protein G at 1:400 have allowed for better detection of bat Ab in your assays?

Though protein G was the second reactive antibody, a specific secondary antibody is much preferred. The sensitivity of anti-bat IgG was sufficient for us to conclude.

With respect to the initial IFAT methodology (presented in Fig 2A) and used throughout, simply having the negative control consist of untransfected VeroE6 cells and unimmunized serum is not sufficient. Using Fig 2A as an example, the results presented are clear, however additional controls should be added. This includes serum from unimmunized mice bound to transfected cells (all antigens) to show that there is no non-specific binding leading to fluorescence in your assay. And sera from immunized mice bound to untransfected cells will show that there is no non-specific binding of immune sera to VeroE6 cells that do not express hantavirus N antigens. Also, the use of the monoclonal as a positive control against these antigens would be beneficial. As the data are currently presented, these cannot be ruled out.

According to your suggestion, additional controls have been performed and updated in the revised manuscript Fig. 2.

Minor comments:

Line 209: delete “IgG” after Goat in Goat anti-bat IgG

The section has been modified according to your suggestion on page 11, Line 261 in the revised manuscript.

Line 249: the authors state that there is diffused fluorescence in the cytoplasm, which they may be. Why did they not use DAPI to stain the nuclei? This would provide clear evidence that staining is localized in the cytoplasm and is usually standard for these types of IFAT.

According to your suggestion, the use of DAPI helps to differentiate nuclear staining from cytoplasmic expressions. However, the expression pattern and fluorescence in hantavirus NP are evident enough, with a clear distinction between the cytoplasm and the nucleus. Hence, we did not consider DAPI nuclear staining. Again, there are numerous of literature supporting the expression pattern of hantavirus NP.

Line 271: in this section it is not clear what cut-off the authors used for determining what was positive cross-reactivity. In the discussion it mentions a possible 4800 dilution as positive, but it again isn’t clear as this is mentioned as a requirement for Western blot. They should clarify that here and, in the methods, because there are multiple instances of sera reacting at 3200 dilution in Table 1, which might confuse the reader when the authors mention only the ROBV and QZNV cross-reactivity. It also mentions there is “almost” one-way cross-reactivity for ROBV and QZNV, but this is also not clear. Does that mean that 6400 almost falls below the cut-off?

According to your suggestion, a cut-off for cross-reactivity needs to be clearly stated for the dilutions to clarify at what dilution cross-reactivity can be confirmed. We have modified the revised manuscript on page 28, lines 572 - 577.

“The cut-off for cross-reactivity in laboratory-generated antisera according to this manuscript was estimated as 51200 titers. It was also clear that laboratory-generated sera titers were different from field antisera. While 4800 IFAT antibody titers of bat sera were also positive in Western blotting, 51200 IFAT titers from the laboratory-generated antisera were only cross-reactive in IFAT and not in Western blotting. Hence, a 51200 IFAT titer cut-off was used for the 11 antisera generated to estimate cross-reactivity”.

Lines 307-314: the authors us acronyms for the viruses throughout the paper but then switch to virus names in this section. They should keep this consistent throughout.

The section has been modified in the revised manuscript on page 4, lines 115 – 119. And we checked descriptions of virus name throughout manuscript.

Line 396: the low cross-reactivity mentioned here is, I assume, related to the above comment, where some of the samples were positive at 3200. The authors do a good job of explaining why this might be here, but as with the above comment, I think a quick explanation of the chosen cut-off in the results and methods would make things clear earlier in the paper to the reader.

The section has been modified in the revised manuscript as referred to above on page 16, lines 360 -361.

Line 416: this wording is a bit confusing. It should maybe read “IFA antibody titers above 4800”.

The section has been modified in the revised manuscript on page 28, line 574.

Line 428: it should be made clear this is purely speculation, that insectivorous bats may be exposed through a different route.

According to your suggestion, our assertion that “The insectivorous bats may have been exposed to the virus via a different transmission route from the route by which the fruit bats were infected” is pure speculation and is true, that is why we emphasized, “may have been” in revised manuscript lines 642-643.

Line 434: the numbers here, 80 and 97%, are not correct. This is not the positivity rate reported. I assume they mean the 97 and 80 absolute numbers reported in table 3?

The section has been modified in the revised manuscript on page 29, line 606.

The number of positives of the QZNV antigen was similar to that of the BRNV (80 and 97, respectively).

Line 548: the authors state that the study investigated bat- and shrew-borne hantaviruses circulating in Zambia. This is not really the case, and the wording should be changed. There was no active surveillance of bats or shrews carrying virus in the study, only retrospective analysis of serum to examine possible exposures in convenience samples.

According to your suggestion, the referenced line 548 is not correct rather 458. The section has been rectified as: This retrospective study investigated the serological detection of bat- and shrew-borne hantaviruses circulating in Zambia in the revised manuscript on page 30, lines 642-643.

Line 465: I would also change this sentence, as this study does not really clarify the distribution of these viruses in Zambia. First, it does not look at the presence of viruses, or even RNA, so there is no data on the presence of either type of virus, only past exposure across a (long) given time period. Second, there is no data presented on the “distribution” of the viruses or seroprevalence in Zambia, as no data are given on the locations of the animals sampled, only that the animals sampled were from Zambia.

Though there was no viral genetic material detected in this report, this does not completely rule out the presence of some of these viruses. Most of these bats are not migratory, especially the insectivorous bats. Hence, serological tools can equally be used to predict the presence of a pathogen in a study area. We also plan to initiate the detection of genetic materials from fresh samples as described in revised manuscript P31, line 651.

Line 466: all that can be concluded from the data presented is that bats harbor antibodies that are reactive to shrew-borne viruses, not that bats have acquired the viruses. While the nice data presented here suggest minimal cross-reactivity, this was only for 5 or 6 viruses of each virus.

According to your suggestion, we have revised as: It is not known how bats harbor antibodies to shrew-borne viruses. Page 30, lines 642-643 in the revised manuscript.

Line 468: relatedly, this phenomenon should be discussed further, specifically how if we are able to consistently find reactive Ab in bats to “shrew-borne” viruses (or viruses that were initially discovered in shrews), their labelling as “shrew-borne” may be a misnomer. We would need studies to look at the actual prevalence of virus or RNA in bats vs shrews of these viruses.

According to your suggestion, the name shrew-borne may be a misnomer. Yes, we agree. However, not until we have successfully detected the genetic material of these viruses in bats. As stated in the conclusion further studies are needed, that will consider viral genomic detection

Figure 2: Labels for each antigen should be added to Fig 2A.

According to your suggestion, Fig. 2 has been modified in the revised manuscript,.

**Answers to Reviewer #2**

Manuscript Number: **PNTD-D-24-01168,** Sikatarii et al.

Development of a Seroepidemiological tool for bat-borne and shrew-borne hantaviruses and its application using samples from Zambia.

I greatly appreciate your thoughtful and helpful suggestions. I am returning the above manuscript and have made appropriate revisions based on your comments. Our responses to your suggestions are numbered according to your suggestions. Corresponding sites were shown in grey markers in the revised manuscript.

Reviewer #2: Figure 1: Parts B and C could be considered for Supplemental Material. Figure 1 could then be expanded by a comparison among the N proteins (identity percentage).

According to your suggestion, Fig 1A has been modified in the revised manuscript. An identity percentage table was inserted.

Figure 2: The authors should label the different IFAs in part A. For part B, the authors should indicate the location of the expressed rN. Please explain the multiple bands of higher and lower molecular weight reacting with the antibodies - degradation or processing products.

According to your suggestion, we modified Fig. 2 with additional control antigens and negative control sera in the revised manuscript.

Table 1: The authors should define and provide the rational for their cross-reactivity cut-off.

According to your suggestion, a cut-off for cross-reactivity needs to be clearly stated for the dilutions to clarify at what dilution cross-reactivity can be confirmed. We have modified the revised manuscript on page 28, lines 572 - 581.

The cut-off for cross-reactivity in laboratory-generated antisera according to this manuscript was estimated as 51200 titers. It was also clear that laboratory-generated sera titers were different from field antisera. While 4800 IFAT antibody titers of bat sera were also positive in Western blotting, 51200 IFAT titers from the laboratory-generated antisera were only cross-reactive in IFAT and not in Western blotting. Hence, a 51200 IFAT titer cut-off was used for the 11 antisera generated to estimate cross-reactivity. Generally, a 4-fold difference in antibody titer showed independent antigenicity. To evaluate true antigenic cross-reactivities, naturally infected animal sera are required. In this study, we did not detect cross-reactivity from bats sera captured in Zambia. Further careful study is needed to evaluate the cross-reactivity of bat-borne and shrew-borne hantavirus antigens.

Figure 3: As a control the authors should have considered transfection with a plasmid expressing an unrelated protein or just ‘empty’ plasmid DNA rather than non-transfected cells.

According to your suggestion, unrelated or empty plasmid DNA could be used rather than untransfected cells. Further controls were performed with empty pCAGGS plasmid DNA. Hence, the results have been modified in Fig. 3.

Serological screening: Are the 107 sera positive to both mixed antigens true co-infections. If yes, is there a pattern or a random distribution regarding individual antigens? This should be better described.

As you pointed out, the combination pattern of sera showing multiple positives was not significant. It looks completely random. So, we added these results in the results section on page 26, lines 512 - 520 in the revised manuscript.

Figures 4 & 5: These figures may need some redesigning as they interrupt the text flow.

According to your suggestion, Figures 4 and 5 have been modified to make the bat sera dilution table stand alone in the revised manuscript. Pages 19 -23.

Make sure that the figure legends contain all important information.

According to your suggestion, we checked figure legends throughout manuscript and modified.

Reviewer #2: The manuscript by Sikatarii and colleagues describes the development of seroepidemiologic tools for bat-borne and shrew-borne hantaviruses. Recombinant nucleoproteins from different bat- and shrew-borne hantaviruses were expressed in cells and used to screen bat sera from Zambia. The authors conclude that bats in Zambia are likely exposed to both types of hantaviruses.

General Comments:
(1)	The detection of serological responses in bats against shrew-borne hantaviruses is surprising and likely cannot be easily explained. Is there evidence others than serology that would support this finding such as genome detection by RT-PCR or virus isolation? The findings should be discussed with caution here.

Unfortunately, there is no molecular epidemiological information of hantavirus from Zambia. We did not have the opportunity to analyze fresh samples. It was a big surprise for us to find African bats with antibodies to the Asian Shrew-derived virus antigen. In any case, we believe that further investigation is necessary. We discussed this on page 31 lines 651 - 653, in the Discussion section.

(2)	An ELISA platform would likely be easier and simpler than an IFAT/westernblot. The authors should provide their rational for their choice and discuss the ELISA option.

IFAT can test with a small amount of serum or plasma. This is useful because many bats weigh less than 10g. Both ELISA and IFAT have their own advantages. It would be good to be prepared to choose one to depend on the situation. In discussion section, lines 654-662 were inserted.

(3)	The low level of cross-reactivity among the different hantavirus N proteins is surprising. Why is that? What is the sequence identity of the different N proteins in percentage? This should be described/discussed, and the comparison be presented as a table in Supplemental Material.

Per your suggestion, the section has been modified in the revised manuscript on page 9. However, considering the phylogeny distance and amino acid identity, the cross-reactivities among used viruses were expected to be low. An identity table was created for comparison and shown in Fig. 1A.

**Answers to Reviewer #3**

Manuscript Number: **PNTD-D-24-01168,** Sikatarii et al.

Development of a Seroepidemiological tool for bat-borne and shrew-borne hantaviruses and its application using samples from Zambia.

I greatly appreciate your thoughtful and helpful suggestions. I am returning the above manuscript and have made appropriate revisions based on your comments. Our responses to your suggestions are numbered according to your suggestions. Corresponding sites were shown in green markers in the revised manuscript.

Reviewer 3

The manuscript “Development of a seroepidemiological tool for bat-borne and shrew-borne hantaviruses and its application using samples from Zambia” by Sikatarii et al. is an interesting paper that describes the development of a serological testing algorithm and suggests that “shrew-borne” hantaviruses more commonly infect bats than “bat-borne” hantaviruses. The sampling appears to be opportunistic from previously available samples so is lacking the numbers needed for extensive analysis (this is not necessarily a negative, but the bat capture study does not appear to have the statistical power for confident modeling) but is convincing to show the utility of the seroepidemiological tool. There are several confusing or missing information in the paper which needs to be clarified prior to submission (see specific comments below).

In the Abstract and author summary.

Line 38- please clarify which positive control antiseta (i.e. mouse and rabbit) were generated.

Per your suggestion, the section has been modified in the revised manuscript on page 2, lines 38 - 40.

“Antisera against the Nus-tagged rN fusion proteins of these viruses (mouse anti-Brno, Dakrong, Quezon, Robina, Xuan Song, Asama, Cao Bang, and Nova, while rabbit anti-Altai, Seewis and Thottapalayam) were also generated.”

Line 39 – please clarify that the authors evaluated the reactivity on mixed transfections and on singly transfected Vero cells. Perhaps also include a justification as to why vero E6 cells were chosen as they are poorly transfectable.

Per your suggestion, the revised manuscript has been modified on page 2, lines 43- 46 to provide more explanation.

Line 42 – the word altogether means completely or totally, where are the authors appear to mean “all together” as in at the same time.

Per your suggestion, the section has been modified in the revised manuscript on page 2, lines 43-46.

For serological screening of bat samples, 5 rNs of the bat-borne viruses were expressed together in a single transfection protocol. Similarly, 6 rNs of shrew-borne viruses were expressed as such. Reactivities of the mixed antigen system were also examined across the singly transfected Vero cell lines to ensure that all antigens were expressed.

Lines 56-58 – I am unsure what the authors are trying to say here. I do not know howthe sentiment of “more data has been generated than there are people to make meaningful conclusions from” is relevant to a paper which provides more data and a tool to generate additional data. I recommend removing it.

Per your suggestion, the section has been modified in the revised manuscript on page 3, lines 68 - 70.

“Advancements in scientific research tools in areas of emerging and re-emerging zoonotic diseases of public health importance are crucial to understanding the epidemiology of these diseases.”

Line 64 – Remove the “therefore” from this line.

Per your suggestion, the section has been modified in the revised manuscript on page 3, line 76.

Line 65 – Remove the word “some” and change the word “bats” to “bat”

Per your suggestion, the section has been modified in the revised manuscript on page 3, line 77.

Line 66 - Replace the “as well as” with the word “with”

Per your suggestion, the section has been modified in the revised manuscript on page 3, line 77.

Line 68 – Remove “the relevant”

Per your suggestion, the section has been modified in the revised manuscript on page 3, line 79.

In the main body of the paper:

Line 79- Change from “Apart from rodent-borne hantaviruses…” to “Apart from in rodents…”

Per your suggestion, the section has been modified in the revised manuscript on page 4, line 101.

Line 94 – Is there a reason Quezon and Robina viruses instead of QZNV and ROBV? If not change it back for consistency with the rest of the paper.

Per your suggestion, the section has been modified in the revised manuscript on page 4, line 115-page 5 line 119.

Lines 116-118 – This sentence is confusing please change it to something like “ Additionally, due to a lack of available tools, only a few small scale studies have been reported on the potential for bat- and shrew-borne hantaviruses to infect humans.”

Per your suggestion, the section has been modified in the revised manuscript on page 5, lines 140-141.

In the materials and methods section the source of materials is not consistent, sometimes it is just the company name, sometimes it’s the company name, city, state, country, sometimes the product number is specifically listed). Make this consistent with PLoS reporting requirement.

Per your suggestion, the section has been modified throughout materials and method section in revised manuscript.

Lines 144-160. Can the authors state how many wells were considered for each positive or negative. E.g. was each sera applied to 3 wells of the slide glass? Was it 4 wells? The N value is missing. The western blots were loaded in quadruplicate which makes me suspect it was done in n=4 throughout but this is not documented.

Similarly, were these experiments just done once or were they repeated with data from a representative repeat shown?

Per your suggestion, the section has been modified in the revised manuscript on page 7, lines 192-193.

“Triplicates of each serum were applied on 3x8, 24-well glass slides for all analyses.”

For the western blotting section please add the anti-Nus western blot data. This is mentioned previously in the preparation of antisera to rN proteins, however the western blot methods are missing.

Per your suggestion, the section has been modified in the revised manuscript in lines 202 – 205.

Line 252 – do you have any more specific data than “approximately 10% to 20%” was it 15±5% for an N=96?

Per your suggestion, the section has been modified in the revised manuscript on page 12, line 318-320. “IFAT demonstrated that the transfection efficiency of each plasmid vector was approximately 10% to 20% (The total expression in three wells was compared to the unexpressed cells in the same well. Hence, the population of expressed cells was estimated at 15±5%)”

Line 253-254 – in the materials and methods the authors state that they used the anti-nus antibodies for determination of expression of recombinant antigens but in the results and in the figure it appears as though the authors are using the mouse/rabbit sera for detection of the antigen. Can you please clarify which sera were used for the assessment of protein expression.

Per your suggestion, the section has been modified in the revised manuscript on page 8, line 202-205.

“The anti-nus antibody was used to confirm the preliminary expression of the various proteins in BL-21 cells before proceeding to produce the respective antisera by using HRP-conjugated anti-mouse (Jackson ImmunoResearch, USA) as secondary antibody.” Western blot patterns were shown in upper panel of Fig. 2B.

Line 271 QZNV was accidentally written as QUZV and needs to be changed back.

Once again write in the N that these titrations are based on.

Per your suggestion, the section has been modified in the revised manuscript on page 4, line 111.

Lines 291-292 – Not sure they product numbers are listed here and nowhere else in the paper.

Per your suggestion, the section has been modified in the revised manuscript, page 11, lines 261-262.

Lines 302-304 – Can you please explain why you only tested further the 401 that were positive for either the bat or shrew borne hantaviruses but not both? This part is confusing as it would be interesting to see information for these if it is available.

The 401 positives were for either bat or shrew positive which means the 107 that were both positive for bat and shrew were also part of the overall 401 positives.

Table 2 – The math is off for this whole table. For example a total of 986 Ei helvum were tested, 270 were positive to either antigen, 81 to both antigens and 716 were negative i.e. 270 positive (for either or both) and 716 negative. However columns next to it clearly state that 156+195 =351 total bats were positive for bat or shrew antigen of which 81 were positive for both so 270 were positive for either antigen but not both. However 986-351 is only 635 negative samples instead of 716. So which number is incorrect or which title is incorrect? Please fix throughout the table.

Per your suggestion, Table 2, specificities column has been modified in the revised manuscript. The 351 total positives for the mixed systems rather consist of “either antigen” and “both antigens.” Hence, bat only is calculated as 156–81=75. Likewise for the others. For better understanding number of bat-antigen only positive and shrew-antigen only positives were added.

Lines 306-340 – Typical reaction patterns of bat serum in IGAT and western blot

Why were these bats chosen to be representative? Are they from different species or all from one species? To me ZFB09-ZFB14 suggests “Zambian fruit bats” caught in 2009 through 2014 with the –xx being the number that was caught, however this leads to more questions that this study is willing to or able to explain. Please remove this nomenclature.

Per your suggestion, the section has been modified in the revised manuscript on page 19, lines 449-3456. Representative samples were selected based on the amount of serum available for Western blotting. Ten samples were selected.

“Generally, the reaction patterns of the bat serum sample on IFAT and WB showed varying degrees of reactivity to the respective rN proteins. For example, ZFB06-11 (ZFB=Zambian fruit bat, 06=year, 11=sample number) showed reactivity to DKGV and XSV antigens on individual IFAT but was reactive to XSV only on western blotting assay. Similarly, ZFB14-151 showed reactivity to both BRNV and ROBV on individual IFAT; however, only BRNV was reactive on western blotting. Other reaction patterns indicated mixed infection, as seen in ZFB11-40, where individual antigens showed reactivity to both BRNV and CBNV antigens and were detected on western blotting assays.”

Figure 4. B Do the authors have data for the other bats or was titration only done on these 6 bats? If so why were these 6 bats chosen and not the other 395 bats? This really should be a stand alone table and not part B of figure 4.

D, E and F. Why are these here? Figure 4 and Figure 5 show the same things just with different antigens. Please remove these as they do not add anything beyond Figure 4A and C.

Per your suggestion, the section has been modified in the revised manuscript. The table of antibody titers has been moved to Table 3. The titration was done for only those selected bat sera. We decided so because the analysis was done to confirm the system we developed—also, the screening was not to report on active surveillance. We may consider total titration for active surveillance purposes. Fig. 4A showed IFAT pattern of Mix antigen-bat. Based on screening results we selected sera for further confirmation tests. IFAT and western blot patterns of selected sera were shown in Fig. 4B, C, D, and E. Figure 5 showed similar screening and confirmation process based on Mix antigen-shrew.

Line 345 the 25 is a typo here according to Table 3 this should be 15.

We apologize caress mistype, the “25” has been modified in the revised manuscript on page 23, line 468.

The bat sera showed reactivity to more than 1 hantavirus (71 of the 201 for bat borne and 7 of the 307 for the shrew borne) can you add another column which states which were the bat positive sera.

Per your suggestion, the Table 2 has been modified. Two additional columns have been added to describe bat-only and shrew-only.

Lines 351 to 373. This section seems weak in its predictive power due to the lack of consistent numbers in samples.

Furthermore the text lacks any numbers, can the authors provide some data in this paragraph instead of just saying it is significant. For example Figure 6b appears to show that 11% of ALTV positivity in R. aegyptiacus compared to 6% ASAV is significantly different. But what does mean in terms of absolute numbers 41/370 for ALTA vs 22/370 for ASAV? Please include some numbers in the text.

I’m not sure what Figure 6 is trying to prove due to the inherent issues of comparing between bat species.

I would remove this section because it does not add anything to what the main point of this paper seems to be i.e. the IFAT using rNs, but not that bat capture field studies from 2008-2014 didn’t capture enough bats to reach statistical significance for a certain bat species.

Per your suggestion, the section has been modified in the revised manuscript on page 24, from line 488 to page 25 line 499.

“Next, the seropositivity rates of individual antigens within each species were compared (Fig 6B). The reaction profiles of fruit bats to the antigens of bat-borne hantaviruses were similar as seen *Eidolon helvum* with BRNV recording 7.9% (78) as the highest and 6.6% (65) for QZNV as the second highest. A similar pattern was observed in the *Rousettus aegyptiacus* species, as shown in Fig 6B. In contrast, reactivity to the antigens of the shrew-borne hantaviruses of *R. aegypticus* showed a significantly lower seropositivity rate to the CBNV antigen recording 1 positive out of 370 sera. In addition, a significant difference was detected between the seropositivity rates of the ASAV 6.2% (23) and ALTV 11% (41) antigens. Due to the inherent issues of nonequal sample sizes, comparisons between bat species were not recommended. The secondary antibodies used in this study were not guaranteed to react equally with all bat species tested; therefore, no cross-species comparative analyses were performed.”

Lines 375- 382 should be moved to the individual antigen screen section after line 350 as they seem to be a continuation of this section not a standalone section.

According to your suggestion, the section has been modified in the revised manuscript on page 26, lines 512 - 520 but we still maintained the test under statistic in order not to distort the test flow.

Lines 405-409. Is there any data for this in the results or mention of it in the materials and methods that I am missing? This whole section is not explained at all, why or how this was done. Remove it or explain it in methods, supplemental methods or results.

Thank you for your suggestion included this in the methodology under “Preparation of antisera to rN proteins” in Materials and Method section page 7-8 in revised manuscript. Results of rNs expressed with NUS-tagged protein were inserted in Fig. 2B.

Line 414 – Replace “solely” with “individually”.

Per your suggestion, the section has been modified in the revised manuscript on page 28, line 568.

Line 415 – please add this data i.e. how many samples reactivity to 2 or 3 samples in Table 2.

Per your suggestion, Table 2 in the revised manuscript on page 18 has been modified.

Line 416 – 4800 IFA points is an impressive number but this information on the N of each analysis is lacking in the methods and results. Please add it there not in the discussion.

Per your suggestion, the revised manuscript has been modified on page 7, line 161.

Triplicates of each sample were applied on 3x8, 24-well glass slides for all IFA analyses as shown in page 7, lines 192-193.

Line 434 Why is the total number from table 2 now listed at 80% and 97%? I was under the impression that 97/1764 samples were positive for BRNV and 80/174 were positive for QZNV. Is this what the authors mean?If it was 80% and 97% that would be very high but the data in previous sections does not seem to support this, unless the authors can explain where 80% and 97% come from.

The section has been modified in the revised manuscript on page 29, line 605.

“The number of positives of the QZNV antigen was similar to that of the BRNV (80 and 97, respectively).”

General point – perhaps the authors want to provide a paragraph as to how they envision their tool will assist the further research that is required to advance the understanding of the epidemiology of hantaviruses.
Per your suggestion, the section has been modified in the revised manuscript on page 30, lines 647 – page, 31653 and page 31, 662-664

Reviewer #3: There needs to be information on group sizes, repeats, how many times things were done. I have no idea what the n is for any of the assays. Based on the western blots I assume the n=4 but this needs to be stated.

Per your suggestion, the section has been modified in the revised manuscript on page 7, lines 192-193.

The source of materials needs to be harmonized e.g. (company name) vs (company name, city, state, country) throughout.

The source of materials throughout manuscript has been modified according to your suggestion

Some methods are missing e.g. how they performed the Nus tag western blot as opposed to the bat sera western blot.

Western blotting was performed by following the usual conventional methods as partly described in the methodology on page 8, line 202-205 as well as page 11, lines 261 - 262.
--------------------

Reviewer #3: Tables have to be updated as the math or the titled does not add up e.g. Table 2 Eidolon helvum row mix-antigen-shrew needs to state that the positive to both is a part of positive to either, not in addition to the 270.

As you pointed out, I think there was a lack of precision regarding the specificity in Table 2.　So, the Table 2 has been modified to show details of specifics.

Figure 4 and 5 need to be made more consistent with respect to how many sections they have and the sample numbers need to be modified to not be what they are called in the author's spreadsheets.

Per your suggestion, the IDs in Figures 4 and 5 are elaborated in the revised manuscript.

The final section has no title and should be combined with the single rN expression section.

The section has been modified according to your suggestion by inputting a befitting title on page 26, line 512

Not sure how the statistical analysis is done with respect to how the p values were calculated and there are no error bars.

sample numbers have to be stated on figures.

The statistical analysis on page 24 in the revised manuscript was done by comparing the reactivity within species since it was impossible to compare between species because of the inherent issue of unequal sample size. The explanation for the Figures has been modified in the revised manuscript on page 25, lines 497 - 498.

Reviewer #3: The the conclusions that this is a useful tool for seroepidemiological studies is well supported, however the statistical analysis appears to be weak likely due to the fact that the sample for this study wasn't intended for this tool and may not have the required numbers for appropriate statistical power. This is expected as this was an opportunistic use of already available samples that were collected for another purpose. This in no way invalidates the publishing of the tool which is the real strength of this paper.

Thank you for your statement “This in no way invalidates the publishing of the tool which is the real strength of this paper.”

Editorial and Data Presentation Modifications?

Reviewer #3: I would like to see another paragraph that discusses the actual tool in the discussion as this is the big development of this paper as far as I see it. 

According to your suggestion, a paragraph has been added to the revised manuscript elaborating on the strength of the developed tool on page 31, lines 654 - 664.

The figures need to be made more consistent and the table needs to be removed from figure 4 and set up as a standalone table.

According to your suggestion, the bat sera titeration table has been moved to table 3 in the revised manuscript.
